# Stable isotopes reveal diet shift from pre-extinction to reintroduced Przewalski’s horses

**DOI:** 10.1038/s41598-017-05329-6

**Published:** 2017-07-20

**Authors:** Petra Kaczensky, Martina Burnik Šturm, Mikhail V. Sablin, Christian C. Voigt, Steve Smith, Oyunsaikhan Ganbaatar, Boglarka Balint, Chris Walzer, Natalia N. Spasskaya

**Affiliations:** 1 Research Institute of Wildlife Ecology, University of Veterinary Medicine, Vienna, 1160 Vienna, Austria; 2 Norwegian Institute for Nature Research – NINA, P.O. Box 5685 Sluppen, NO-7485 Trondheim, Norway; 3 Zoological Institute RAS, Universitetskaya nab. 1, 199034, Saint-Petersburg, Russia; 4 Leibniz Institute for Zoo and Wildlife Research, Alfred-Kowalke-Straße 17, 10315 Berlin, Germany; 5 Konrad-Lorenz Institute of Ethology, University of Veterinary Medicine, Vienna, Vienna, 1160 Austria; 6 Great Gobi B Strictly Protected Area Administration, Takhin Tal, Gobi Altai Province Mongolia; 7 Department of Zoology, School of Biology and Biotechnology, National University of Mongolia, 14200 Ulaanbaatar, Mongolia; 8 Zoological Museum of Moscow Lomonosow State University, Bolshaya Nikitskaya Str. 6, 125009 Moscow, Russia

## Abstract

The Przewalski’s horse (*Equus ferus przewalskii*), the only remaining wild horse within the equid family, is one of only a handful of species worldwide that went extinct in the wild, was saved by captive breeding, and has been successfully returned to the wild. However, concerns remain that after multiple generations in captivity the ecology of the Przewalski’s horse and / or the ecological conditions in its former range have changed in a way compromising the species’ long term survival. We analyzed stable isotope chronologies from tail hair of pre-extinction and reintroduced Przewalski’s horses from the Dzungarian Gobi and detected a clear difference in the isotopic dietary composition. The direction of the dietary shift from being a mixed feeder in winter and a grazer in summer in the past, to a year-round grazer nowadays, is best explained by a release from human hunting pressure. A changed, positive societal attitude towards the species allows reintroduced Przewalski’s horses to utilize the scarce, grass-dominated pastures of the Gobi alongside local people and their livestock whereas their historic conspecifics were forced into less productive habitats dominated by browse.

## Introduction

Captive-breeding is often the last resort in efforts to save endangered species, and has been instrumental in saving a handful of species from extinction and returning them to the wild^1^. One of these species is the Przewalski’s horse (*Equus ferus przewalskii*), the only remaining wild horse within the equid family^2, 3^. However, concerns have been raised that captive breeding of endangered wildlife will change morphological, behavioral, or genetic traits ultimately compromising fitness or altering a species functional role in the ecosystem^4–6^. On the other hand, we are also increasingly trying to restore species in a world where ecological conditions have changed or are changing^7^. Finding a suitable baseline against which to assess the ecology of a reintroduced species is not trivial, particularly when dealing with rare or “extinct in the wild” species which were already subject to anthropogenic pressures even in historic times^8^.

**Figure 1:**
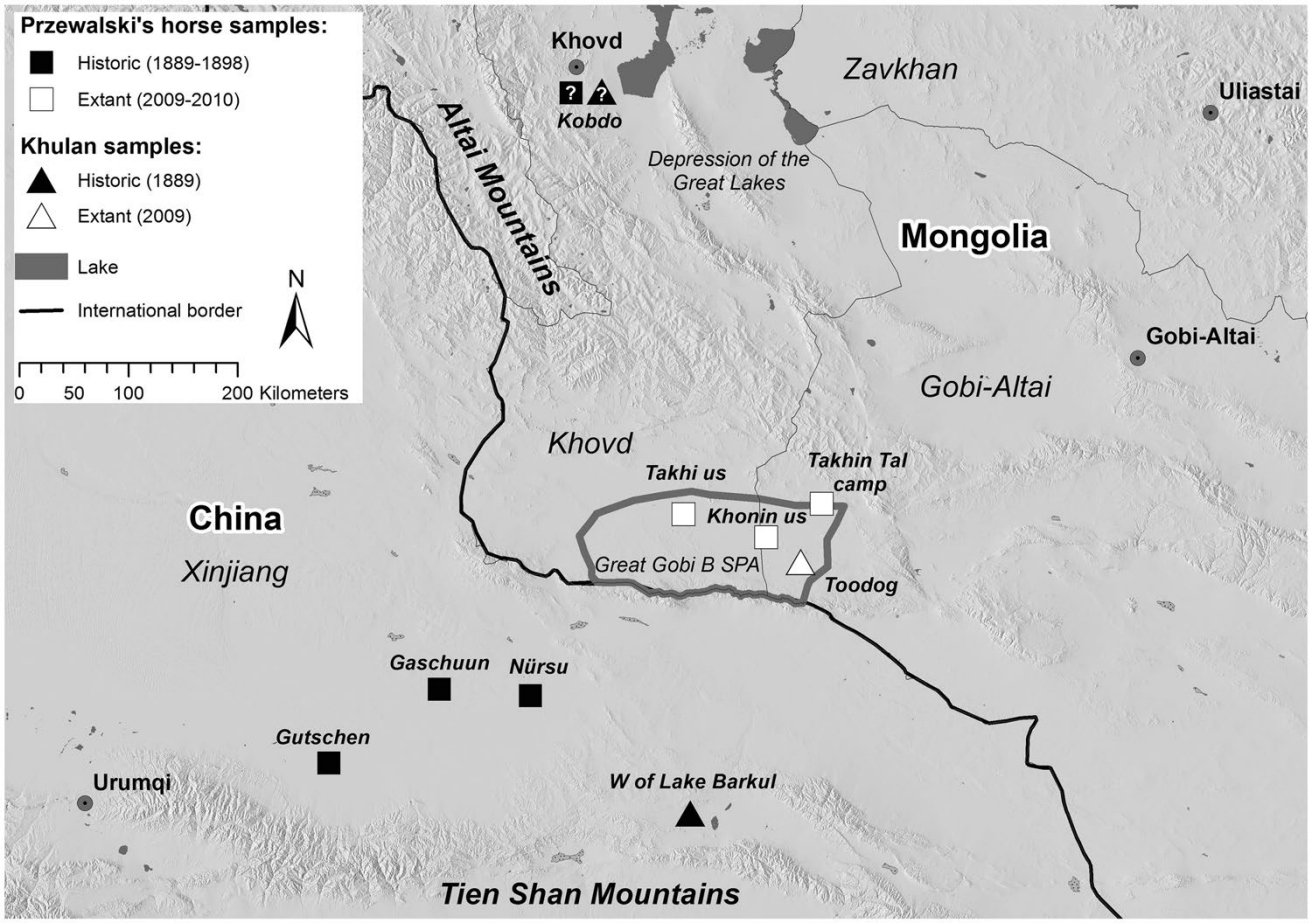
Sampling locations and approximate extent of the Przewalski’s horse distribution in the late 19^th^ and early 20^th^ century in what is today’s *Xinjiang* Uyghur Autonomous Region in northwestern China and Khovd and Gobi-Altai provinces in southwestern Mongolia.? = The exact location of the two historic samples from “Khovd province” (Kobdo) are not known and they may originate from the Depression of the Great Lakes or today’s Great Gobi B SPA. Figure generated in ArcGIS 10.1 (ESRI, Redland, CA, USA, http://www.esri.com/).

By the time the Przewalski’s horse became known to the western world in 1881, it had already become restricted to the most remote areas of the Dzungarian Gobi in what is today’s northwest China and southwest Mongolia (Fig. 1, Table 1). The Przewalski’s horse and its close relative the Asiatic wild ass (khulan, *Equus hemionus*) were targeted as game, persecuted as grazing competitors, and displaced by agriculture^9^. By the late 1960s, the Przewalski’s horse was extinct in the wild and the khulan had been displaced from the steppe and become entirely confined to the Gobi regions^10, 11^. In 1992, the first captive bred Przewalski’s horses were returned to the Mongolian Gobi, where some 20 years previously the last wild horses had been observed^2^ (for history of Przewalski’s horse discovery, extinction and reintroduction see Table 1). The Gobi is still considered part of one of the largest, intact dryland grazing system in the world and has been shared by far ranging ungulates and semi-nomadic livestock herders for millennia^12, 13^.

**Table 1:** Timeline for Przewalski’s horses - discovery, extinction in the wild, and reintroduction. For references see Supplementary Information S1.

• First written accounts of Przewalski’s horses by the Tibetan monk Bodowa around 900 ADS1.
• John Bell (1691–1780), a Scottish doctor in the service of Tsar Peter the Great from 1719–1722, apparently observed the species in present-day Tomsk oblast in SiberiaS2.
• Colonel Nikolai M. Przewalski (1839–1888), a Russian geographer and explorer in the service of Tsar Alexander, obtained the skull and hide of a horse in 1878 which had been shot some 80 km north of Gutschen (Quitai) near today’s Chinese–Mongolian borderS3.
• The skull and hide were examined by Iwan S. Poliakov (1847–1887) at the Zoological Museum of the Academy of Sciences in Saint Petersburg. He concluded that the remains were those of a wild horse which he named *Equus przewalskii* S4.
• From 1899–1903 Friedrich von Falz-Fein and Carl Hagenbeck successfully captured Przewalski’s horses in the Dzungarian Gobi and the Depression of the Great Lakes and transported them to Europe. In total, six transports brought 54 individuals to EuropeS5,S6,S7.
• The last records of a Przewalski’s horse in the wild were reported in the late 1960s from today’s Great Gobi B Strictly Protected Area (SPA) in the Dzungarian Gobi, south-western MongoliaS8,S1.
• The species survived in captivity due to breeding based on 12 wild-caught individuals and as many as four domestic horse foundersS9. In 1959 the International Przewalski’s Horse Studbook was createdS10,S11.
• In 1996 the Przewalski’s horse was officially classified as “Extinct in the Wild” by the IUCN Red List of Threatened Species. However, in 1992 the first captive bred Przewalski’s horses had already returned to Mongolia for reintroduction. In 2008 the Przewalski’s horse was reclassified as “Critically Endangered” and in 2011 as “Endangered”S12.
• In 2013 the reintroduced population in Mongolia numbered > 400 animals at three different sites, including Great Gobi B SPA in the Dzungarian Gobi. Further reintroduction initiatives are under way in China and Russia and are planned for KazakhstanS13,S14,S15.

Very little behavioral or ecological data on Przewalski’s horses prior to their extinction in the wild are available. That which exists, comes from a limited number of written historical sources^14, 15^ or oral accounts of the last eye witnesses^16^. There are few remaining museum samples of specimens collected in the wild, but these constitute the only additional material available to reconstruct the ecology of Przewalski’s horses prior to extinction^17^. Stable isotope analysis of animal tissue has become a powerful tool to draw inferences about various aspects of a species’ ecology^18^. It is particularly useful to address and compare feeding ecology and can make use of a wide variety of samples from ancient, historic, and extant specimens^19, 20^. In equids, tail hair grows continuously and after formation is metabolically inert, hence constituting a valuable time series archive when sequentially cut and analyzed^21–23^.

We used stable isotope analysis of tail hairs to compare multi-year isotopic diet seasonality and dietary niche characteristics of historic (pre-extinction) and reintroduced (extant) Przewalski’s horses from the Dzungarian Gobi and for comparison did similar analysis for historic and extant sympatric khulan. We reveal a dietary shift from being a mixed feeder in winter and a grazer in summer in the past, to a year-round grazer nowadays, which is best explained by a release from human hunting pressure.

## Results

### Seasonality in the diet of historic but not reintroduced Przewalski’s horses

**Figure 2:**
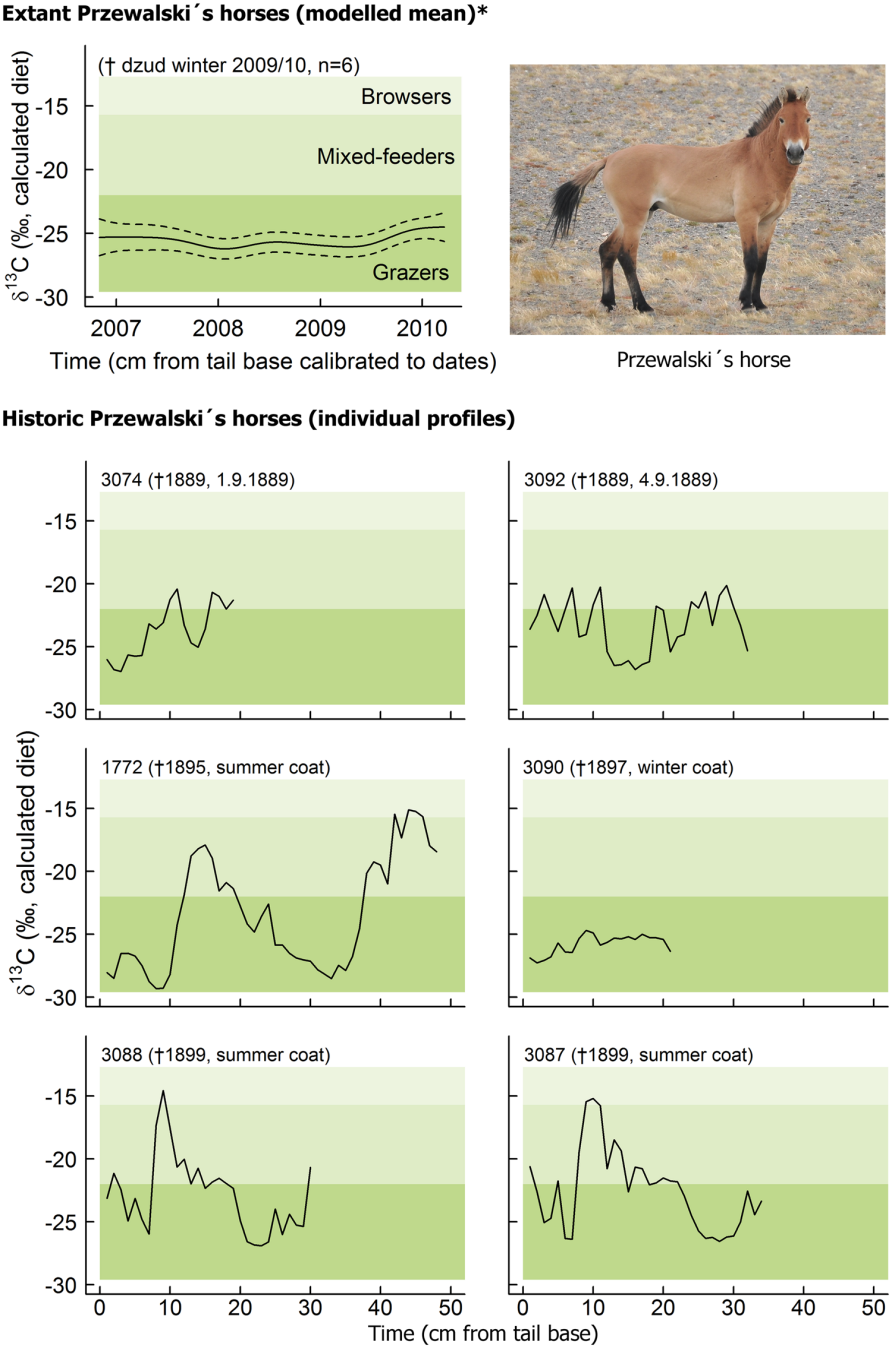
Carbon isotope (*δ* ^13^C_diet_) profiles of reintroduced, extant and historic Przewalski’s horses in the Dzungarian Gobi. Photo: P. Kaczensky.

The tail hairs of five of the six historic Przewalski’s horses showed a clear seasonality in their *δ* ^13^C_diet_ profiles. The lowest values were well within the ranges suggesting the primary use of C_3_ grasses and forbs typical for grazers (>75% grass), but the highest values extended into the value ranges suggesting considerable use of C_4_ shrubs and semi-shrubs typical for mixed-feeders (25–75% browse) or even browsers (henceforward referred to as “browsing peaks”; Fig. 2).

No seasonality could be observed in any of the six extant Przewalski’s horses, all of which almost entirely stayed within the value range of typical grazers; the same was also true for one of historic Przewalski’s horse. Historic khulan also showed a clear seasonality with low values suggesting grazing and high values suggesting browsing. The pattern was more or less identical to the one previously found in extant animals (Supplementary Fig. S1).

The lack of a precise sampling date did not allow for exact alignment to specific dates in historic samples, but a combination of our examination of coat characteristics and *δ* ^2^H seasonality (see Dryad Digital Repository) suggests that browsing peaks in historic Przewalski’s horses and historic khulan coincided with winter and thus follow the same seasonal pattern as has been observed in extant khulan^24^.

### Broader core dietary isotopic niches in historic than extant Przewalski’s horses

**Figure 3:**
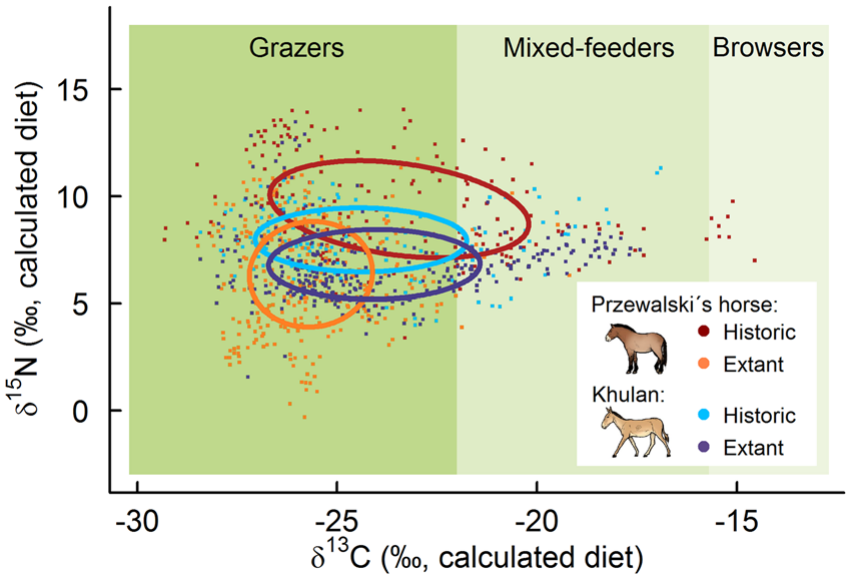
Core isotopic dietary niches of reintroduced, extant and historic Przewalski’s horses and sympatric historic and extant khulan in the Dzungarian Gobi. Artwork: M. van Dalum.

Historic Przewalski’s horses had much broader core dietary isotopic niches than reintroduced ones. The size difference is largely due to a much broader spread along the *δ* ^13^C_diet_ axis; *δ* ^15^N_diet_ values were somewhat higher, but not broader (Fig. 3, Supplementary Fig. S2). With C_4_ browse intake showing only narrow peaks, the core dietary isotopic niche is largely positioned within the range of typical grazers, but clearly extends into the range of a mixed feeder. With one exception, individual core isotopic niches were more consistent in shape and larger in size in historic as compared to extant animals (Supplementary Fig. S3).

Khulan, on the other hand, showed highly similar core isotopic niches in historic and extant individuals, both at the pooled (Fig. 3) and the individual level (Supplementary Fig. S4).

## Discussion

We detected a clear difference in the isotopic dietary composition between historic and reintroduced Przewalski’s horses. Historic Przewalski’s horses switched from a grazing diet in summer to a mixed grass-browse diet in winter, whereas their reintroduced conspecifics graze year round. Sympatric khulan, on the other hand, showed the same seasonality in their isotopic dietary composition in both historic and extant individuals.

The importance of relearning to optimize foraging behavior in highly seasonal, resource poor environments has been previously demonstrated in captive bred Arabian oryx (*Aryx leucoryx*) reintroduced to Oman^25^. However, analysis of forage plant samples from Great Gobi B SPA does not suggest the existence of a nutritional advantage of browse over grasses^26^ and local domestic horses, which have been free-grazing in the region for centuries, also graze in winter^24^. Furthermore, equids are considered typical grazers which include browse in their diet only when lacking alternatives^27, 28^. Hence the diet change between historic and reintroduced Przewalski’s horses suggests a shift from a historic suboptimal diet to a present day more optimal diet, rather than a lack of behavioral adaption among the reintroduced horses.

**Figure 4:**
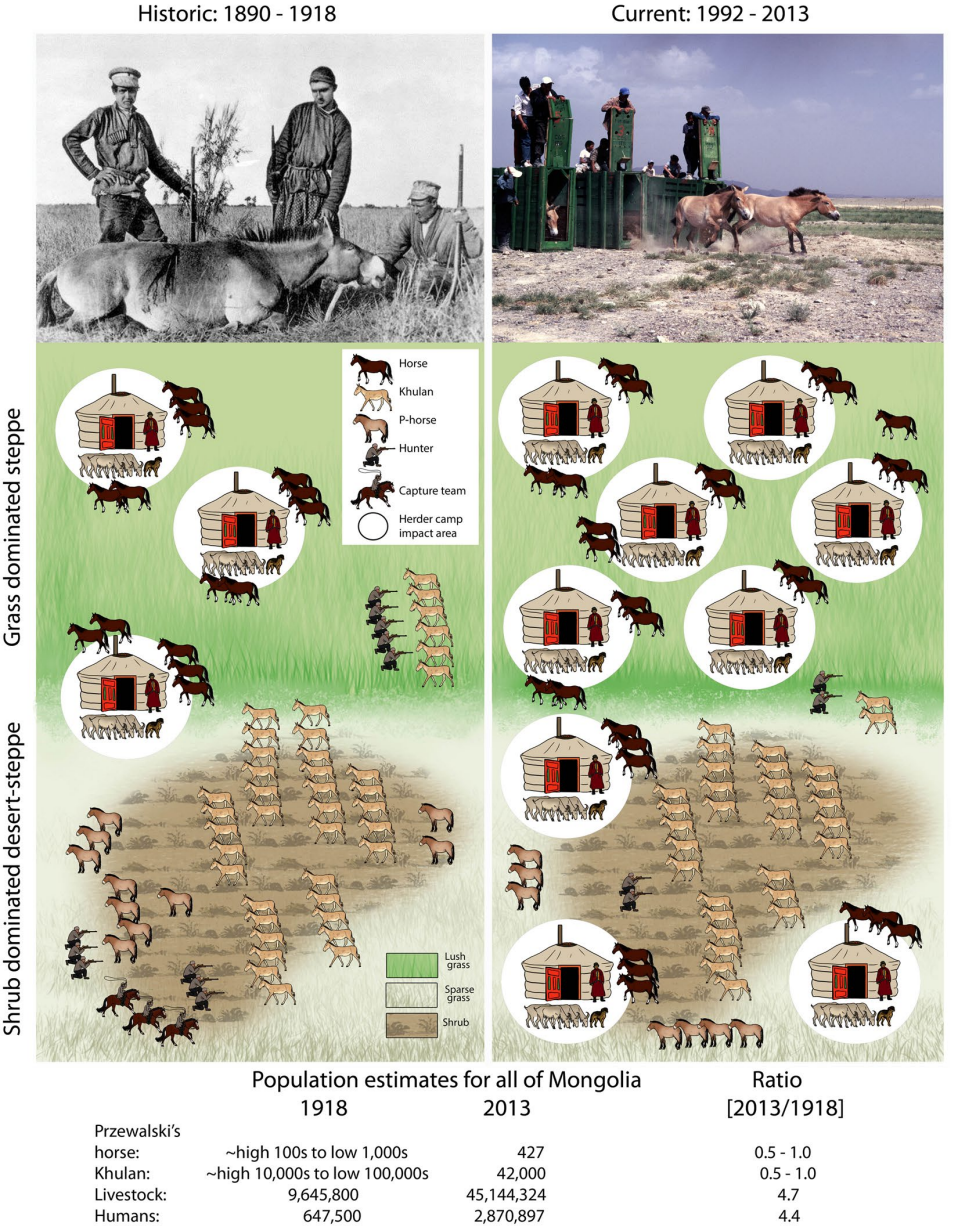
Living conditions of Przewalski’s horses and khulan in the steppe and desert steppe areas of Mongolia in winter past and present. For supporting references see Supplementary Information S5. Photos: Top left source: Grum-Grzhimailo and Grzhimailo 1896, top right: P. Kaczensky, Artwork: M. van Dalum.

The human and livestock population in Mongolia has dramatically increased over the last 100 years (Fig. 4), but Mongolia’s Gobi–Steppe ecosystem still remains one of the largest intact expanses of steppe, desert-steppe and desert habitats in the world^12^. There is also little evidence that C_3_/C_4_ plant ratios are influenced by grazing pressure^29^ and the fact that diets in historic and extant khulan were almost identical further argues against a habitat-induced diet change in Przewalski’s horses.

However, Przewalski’s horses and khulan were heavily hunted without restriction until very recently. As a consequence, the effect of humans has likely long been the defining factor in a “landscape of fear”^30^. Human and livestock presence in Great Gobi B SPA and other parts of the Dzungarian Gobi has always been highly seasonal with local herders leaving in summer and returning in winter^31, 32^. Herder camps are preferably located in grass-dominated habitats and hence wild equids wary of humans will be displaced into suboptimal shrub habitats in winter, likely also causing a shift in diet towards more browse^24^. Habitats with tall shrubs like saxaul may also be preferred as they provide better retreats from (legal or illegal) hunting as wildlife are more difficult to spot and chase there.

Nowadays both wild equids are fully protected, but while attitudes towards the newly returned Przewalski’s horse have seen a dramatic change from that of a game species and pasture competitor in historic times to an iconic national flagship species nowadays^33^, attitudes towards the regionally still numerous khulan remain ambivalent^34^. For conservation reality on the ground, this means *de facto* full protection of the newly returned Przewalski’s horse, but continued and significant levels of illegal hunting and harassment for khulan^35^. As a consequence, extant Przewalski’s horses are rather tolerant of humans, whereas khulan remain wary and avoid the vicinity of herder households^36^ (Fig. 4).

Extant Przewalski’s horses have to share the habitat with more livestock and humans than ever before, yet the release from human predation pressure allows them year round access to the scarce, grass dominated pastures of the Gobi alongside locals and their livestock (Fig. 4). Thus, not the physical environment, but rather the societal environment has changed with a new positive attitude enabling reintroduced Przewalski’s horses to return to the more “natural” state of being a year round grazer. Conversely, browsing in historic Przewalski’s horses in winter is likely indicative of the historic animals being forced into refuge habitats^8^ within the same landscape reintroduced Przewalski’s horses again roam. Our results therefore also represent a cautionary tale about using relatively recent historic states as baselines of the pristine. The human displacement effect on extant khulan, on the other hand, appears unchanged and has been documented for other parts of the Gobi as well^36^. However, khulan seem to be better adapted to exploit low productivity habitats and can also wander further away from water^37, 38^.

Nevertheless, grass dominated habitats in the Gobi are limited and with a growing Przewalski’s horse population and growing livestock numbers, increasing conflicts with local herders have to be expected^32^. Future reintroduction initiatives should preferably aim to reestablish Przewalski’s horses on grass dominated steppes, but will have to carefully balance the gain in habitat quality against the reality of ever higher anthropogenic pressures^39, 40^.

## Material and Methods

### Study area

At the turn of the 19th century, the distribution range of Przewalski’s horses had become confined to the Dzungarian Gobi and the Depression of the Great Lakes in southwestern Mongolia and today’s Xinjiang Uyghur Autonomous region in northwestern China (Fig. 1). By the 1940’s the species was primarily found in todays’ Great Gobi B SPA in Mongolia where the last sightings occurred in 1968. Reintroduction initiatives eventually prioritized the Great Gobi B SPA as a potential release site and the first transport of captive bred Przewalski’s horses arrived in 1992^15, 26^ (for details of the history of Przewalski’s horses from discovery until reintroduction see Table 1). After initial setbacks, the population started to increase and reached 138 individuals by December 2009. The winter 2009–2010 was extremely harsh, killing millions of livestock throughout Mongolia and causing a crash of the Przewalski’s horse population in the Gobi leaving only 49 individuals alive by spring 2010^41^. Reproduction and additional horse transports have since resulted in the recovery of the population to 167 Przewalski’s horses as of December 2016. The Great Gobi B SPA also houses an estimated 5700 khulan, which have had a continuous presence in the Dzungarian Gobi^42^.

The habitats of the historic and extant distribution range of the Przewalski’s horse are characterized by an arid, cold-temperate continental climate with a summer precipitation peak (Supplementary Information S3). The landscape consists of desert-steppe, semi-desert, and desert drylands dominated by Chenopodiaceae (shrubs or semi-shrubs like saxaul *Haloxylon ammondendron*, *Anabasis brevifolia*, and *Salsola* sp.) which follow a C_4_ photosynthetic pathway and Asteraceae (e.g. *Artemisia* sp. and *Ajania* sp.), Tamaricaceae (e.g. *Reaumuria* sp.), and Poaceae (e.g. *Stipa* sp., *Achnatherum splendens*, and reed *Phragmites* sp. forming around larger oases) which follow a C_3_ photosynthetic pathway^43^. Alpine meadows above 2000m are primarily dominated by C_3_ grasses and forbs.

The *δ* ^13^C values of 240 plant samples collected by us in 2012 and 2013 followed a bimodal distribution with mean *δ* ^13^C value of C_3_ plants (13 different grass & forb species) of −25.5 ± 1.3‰ (1σ, n = 198, range from −28.9‰ to −22.7‰) and mean *δ* ^13^C value of C_4_ plants (the two shrubs *H. ammodendrum* & *A. brevifolia*) of −13.5 ± 0.5‰ (1σ, n = 42, range from −12.6‰ to −14.5‰)^24^. This subdivision of the dominant vegetation into C_3_ grasses & forbs and C_4_ shrubs allows the separation of ungulates into being primarily grazers or browsers from isotopic signatures^24^.

The Dzungarian Gobi has long been used by semi-nomadic livestock herders, primarily of Mongol or Kazakh origin. Wherever possible, local herders use the Gobi in winter to profit from the warmer temperatures and lower snow coverage, but leave the hot, dry plains in summer for mountain steppe pastures in the adjacent Tien Shan and Altai ranges^13, 31^. Nowadays population density has increased and land-use intensified dramatically in Xinjiang, but less so in adjacent Mongolia where semi-nomadic pastoralism still remains the key economy in the Gobi region^13, 31^. To our knowledge, there are no records which allow insight into the movement patterns of historic Przewalski’s horses. The high variation in *δ* ^13^C values within individual C_3_ plants^24^ and the substantial overlap in the *δ* ^2^H values of water sources from the high mountains and the Gobi^44^ did not allow us to draw conclusions about potential altitudinal or longitudinal migrations in historic equids.

In the Mongolian part of the Dzungarian basin, Przewalski’s horse groups have annual ranges of 152–826 km², whereas khulan roam over area of 4449–6835 km^2^. However, both species stay in the Gobi year round and both have access to grass as well as shrub dominated plant communities within their annual ranges^37^. Human infrastructure is minimal in the Mongolian part of the Dzungarian basin and no larger roads or railroads dissect the habitat. The only constrain to human and animal movements is the fenced international border between Mongolia and China^45^. Przewalski’s horse and khulan have been fully protected in Mongolia since 1930 and 1953 respectively, but illegal killing of certain wildlife, including khulan, remains a problem^35^.

### Sample collection

We obtained historic tail hair samples from six adult Przewalski’s horses and three adult khulan from museum collections at the Zoological Institute of the Russian Academy of Sciences in St. Petersburg and the Zoological Museum of Moscow Lomonosow State University in Russia (Supplementary Table S1). These historic samples were collected from 1889–1898 in the wild and for 7 out of the 9 samples the location where the animal was killed is known (Fig. 1). For one Przewalski’s horse and one khulan the area of origin is only known as Mongolia’s Khovd province^17^ and thus samples could originate from either the Dzungarian Gobi or the Depression of the Great Lakes. Samples of extant individuals were collected throughout Great Gobi B SPA in 2009 and 2010 (Fig. 1) and analyzed in a previous study^24^. Given the large annual ranges of extant wild equids in the Dzungarian Gobi^37^, historic and extant sampling locations are representative of large surrounding areas.

To allow an approximate alignment of the *δ* ^13^C profiles to dates/seasons (not shown) using the average tail hair growth of 0.57mm/day^21^ a precise collection date is needed, which for historic samples was only available for two individuals (Supplementary Table S1). We thus used overall coat characteristics to at least determine the sampling season (summer coat: May-September, winter coat: November-March; O. Ganbaatar pers. obs.), which was additionally confirmed by the *δ* ^2^H values in the corresponding hair (summer: high *δ* ^2^H values; winter: low values^21^). All tails of the museum specimens were still attached to the hide, making species discrimination straight forward. However, according to W. Salensky^17^ at least one Przewalski’s horse hide had originally been mislabeled “onager [khulan]” and we thus double-checked species identity of all historic Przewalski’s horses using mitochondrial DNA extracted from tail hair samples. The mtDNA analyses in combination with visual inspection confirmed the identification of Przewalski’s horse for all samples (Supplementary Information S4). For the extant samples the exact date of sampling was known and isotope profiles could be assigned to specific dates using methods described by Burnik Šturm *et al*.^21^. All tail hairs were sequentially cut into 1 cm increments. Tail hair lengths ranged from 20–49 cm in the six historic Przewalski’s horses and from 32–63 cm in the three historic khulan (Supplementary Table S2), representing approximately 1–2.5 and 1.7–3.2 years of growth in each species, respectively^21^.

### Stable isotope analyses

We conducted stable isotope analysis at the Leibniz Institute for Zoo and Wildlife Research, Berlin, Germany following methods described in Burnik Šturm *et al*.^21, 24^. The precision of measurements was always better than 0.1‰ for *δ* ^13^C and *δ* ^15^N, and 1.0‰ for *δ* ^2^H values, based on repeated analysis of laboratory standards, calibrated with international standards. In the context of this study, *δ* ^2^H values were primarily used to assign the *δ* ^13^C profiles to the correct seasons (data not shown)^21^. We corrected all *δ* ^13^C results from historic samples by subtracting 1.5‰, the difference in the *δ* ^13^C of the atmospheric CO_2_ between 1900 (−6.8‰)^46^ and 2009/2010 (−8.3‰)^47^, to account for the Suess effect caused by burning of fossil fuels over the last 150 years (see Supplementary Table S2 for mean values of raw isotope values by individual). C/N ratios of all tail hair increments ranged between 2.9 and 3.6, indicating high quality sample preservation^48^.

To convert raw isotope values (*δ* ^13^C_hair_ and *δ* ^15^N_hair_) into diet values (*δ* ^13^C_diet_ and *δ* ^15^N_diet_), we used the dual mixing model of Cerling *et al*.^49^. We obtained C_3_ and C_4_ end–members from the average *δ* ^13^C values (±1 σ) of our 240 plant samples and the diet–hair fractionation factors for horses on a low protein diet^50, 51^: 2.7‰ for C and 1.9‰ for N. We discriminated between grazer (>75% grass), mixed feeder (26–74% browse), and browser (>75% browse) diets based on the definition of Mendoza and Palmqvist^27^.

We used a Bayesian approach to estimate species specific core isotopic dietary niches based on bivariate, elipse–based metrics (for *δ* ^13^C and *δ* ^15^N values) using SIBER implemented in the R package SIAR (Version 4.2)^52, 53^. We assumed normal distribution based on the large sample size (948 data points) and after visual confirmation of a near normal distribution; although the Shapiro-Wilk multivariate normality test (mshapiro.test) did not formally confirm multivariate normality. Core isotopic niche sizes are expressed as the standard Bayesian ellipse area (SEA_B_) in ‰^2^, defined by a subsample containing 40% of the bivariate data. All statistical analyses were conducted in R (version 3.1.1).

### Data accessibility

The raw tail hair isotope data can be accessed from Dryad Digital Repository doi:10.5061/dryad.k98k0.

## Electronic supplementary material

SUPPLEMENTARY DIGITAL MATERIAL
