# Supplementary material for: Stable isotopes reveal diet shift from pre-extinction to reintroduced Przewalski’s horses
Source: Sci Rep. 2017 Jul 20;7:5950. doi: 10.1038/s41598-017-05329-6 (PMC5519547; doi:10.1038/s41598-017-05329-6)
Supplement: Supplementary file 1 — SUPPLEMENTARY DIGITAL MATERIAL [file 41598_2017_5329_MOESM1_ESM.doc]

**SUPPLEMENTARY DIGITAL MATERIAL**

**Stable isotopes reveal diet shift from pre-extinction to reintroduced Przewalski’s horses**

*Petra Kaczensky^1§,2^ , Martina Burnik Šturm^1§^, Mikhail V. Sablin^3^, Christian C. Voigt^4^, Steve Smith^5^, Oyunsaikhan Ganbaatar^6,7^, Boglarka Balint^5^, Chris Walzer^1^, Natalia N. Spasskaya^8^

***Supplementary Information S1:*** *References for Figure 1: Timeline for Przewalski’s horses - discovery, extinction in the wild, and reintroduction.*

S1 Zevegmid, D. & Dawaa, N. Die seltenen Großsäuger der Mongolischen Volksrepublik und ihr Schutz. *Archiv für Naturschutz und Landschaftsforschung* **13**, 87-106 (1973).

S2 Bell, J. *Travels from St. Petersburg, in Russia, to diverse parts of Asia - Volume II* (Printed for the author by R. and A. Foulis, Glasgow, 1763).

S3 Przhevalski, N. M. *Reisen in Tibet und am overen Lauf des Gelben Flusses in den Jahren 1879 bis 1880*. (*C*ostenoble, Jena; <https://archive.org/details/reisenintibetund00przh>*,* 1884).

S4 Poliakof, M. Prejevalsky's Horse (*Equus Przewalskii*, n. sp.). *Proceedings of the Imperial Russian Geographical Society,* 1&2, 1-20 (1881). [In Russian, translated by E.D. Morgan]

S5 Bouman, I. & Bouman, J. The history of Przewalski’s Horse In *Przewalski’s horse -The History and Biology of an Endangered Species* (Boyd L., Houpt, D. A.) *5-*38 (State University of New York Press, Albany*,* 1994).

S6 Hagenbeck, C. *Von Tieren und Menschen* (Vita Deutsches Verlagshaus, Berlin, Germany; <https://ia800205.us.archive.org/32/items/vontierenundmens00hageuoft/vontierenundmens00hageuoft.pdf>, 1909).

S7 Salensky, W. *Prjevalsky's horse (Equus Prjewalskii Pol.)* (Hurst and Blackett Limited, 1907).

S8 Kaszab, Z. New sightings of the Przewalski's horse. *Oryx* **8**, 345-347 (1966).

S9 Bowling, A. T. *et al.* Genetic variation in Przewalski’s horses, with special focus on the last wild caught mare, 231 Orlitza III. *Cytogenetic and Genome Research* **102**, 226-234 (2003).

S10 Mohr, E. *Das Urwildpferd* (A. Ziemsen Verlag, Wittenberg Lutherstadt, Germany*,* 1959).

S11 Volf, J. *Das Urwildpferd* (Die Neue Brehm Bücherei, Band 249, Westarp Wissenschaften, Magdeburg, Germany, 1996).

S12 King, S. R. B., Boyd, L., Zimmermann, W. & Kendall, B. E. Equus ferus. *The IUCN Red List of Threatened Species 2015: e.T41763A45172856*, doi:10.2305/IUCN.UK.20152.RLTS.T41763A45172856.en (2015).

S13 Kaczensky, P. *et al.* Reintroduction of Wild Equids In *Wild Equids - Ecology, Management, and Conservation* (eds Ransom, J. I., Kaczensky, P.) 196-214 (Johns Hopkins University Press, Baltimore, USA, 2016).

S14 Usukhjargal, D. & Bandi, N. Reproduction and Mortality of Re-introduced Przewalski’s Horse Equus przewalskii in Hustai National Park, Mongolia. *J. Life Sci.* **7**, 623-629 (2013).

S15 Xia, C. *et al.* Reintroduction of Przewalski’s horse (*Equus ferus przewalskii*) in Xinjiang, China: The status and experience. *Biol. Cons.* **177**, 142–147 (2014).


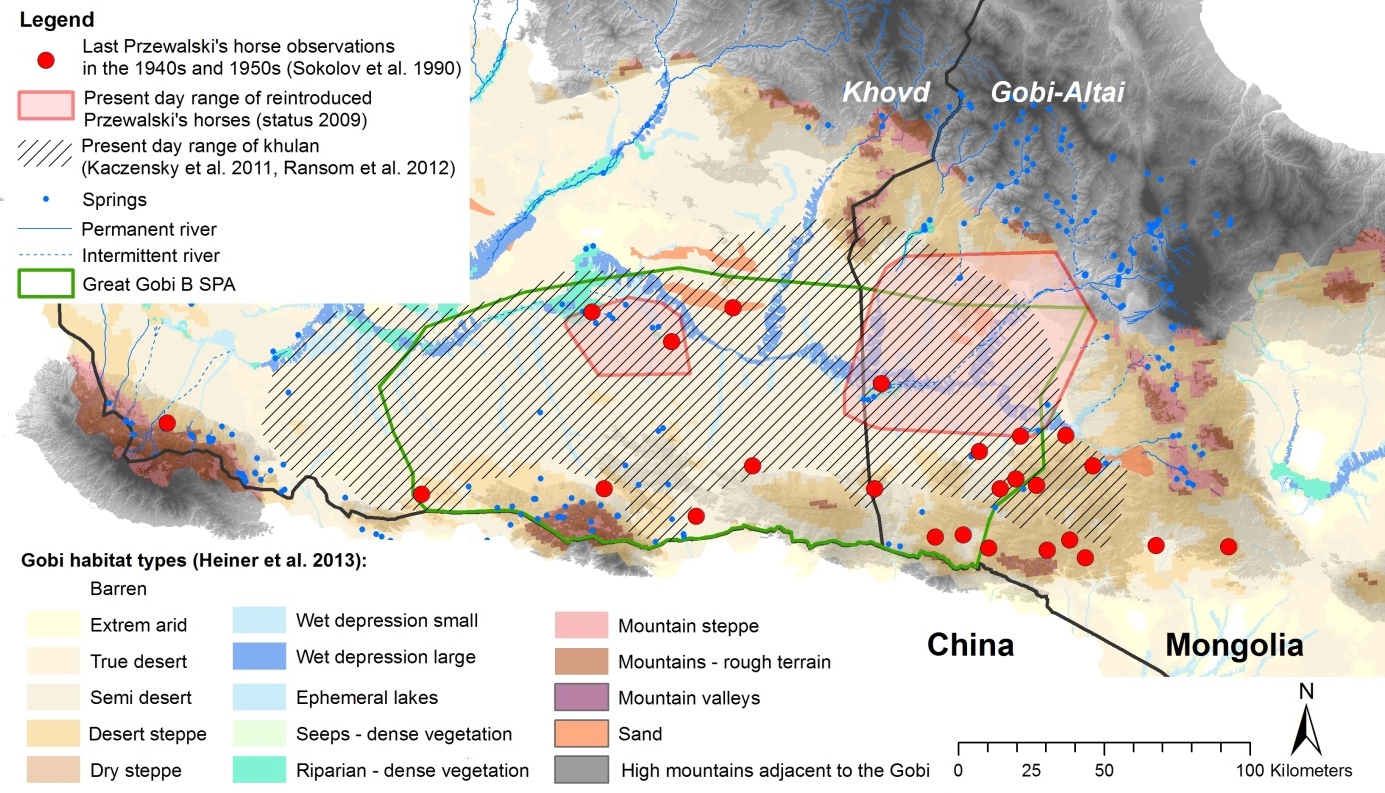
**Supplementary Information S2:** Distribution of i) autochthonous Przewalski’s horses in the 1940s and 1950s based on interviews with eye witnesses (Sokolow et al. 1990) and ii) extant (reintroduced since 1992) Przewalski’s horses and autochthonous khulan in Great Gobi B SPA in the Mongolian part of the Dzungarian Gobi (Kaczensky et al. 2011a,b, Ransom et al. 2011). Figure generated in ArcGIS 10.1 (ESRI, Redland, CA, USA, <http://www.esri.com/>).

**References**

Heiner, M., Bayarjargal, Y., Kiesecker, J., Galbadrakh, D., Batsaikhan, N., Munkhzul, G., Odonchimeg, I., Enkhtuya, O., Enkhbat, D., von Wehrden, H., Reading, R., Olson, K., Jackson, R., Evans, J., McKenney, B., Oakleaf, J., Sochi, K., 2013. Identifying Conservation Priorities in the Face of Future Development: Applying Development by Design in the Mongolian Gobi. The Nature Conservancy, Ulaanbaatar, Mongolia.

Kaczensky, P., Kuehn, R., Lhagvasuren, B., Pietsch, S., Yang, W., Walzer, C., 2011a. Connectivity of the Asiatic wild ass population in the Mongolian Gobi. Biol Conserv 144, 920-929.

Kaczensky, P., Ganbataar, O., Altansukh, N., Enkhsaikhan, N., Stauffer, C., Walzer, C., 2011. The danger of having all your eggs in one basket--winter crash of the re-introduced Przewalski's horses in the Mongolian Gobi. PLoS ONE 6, e28057.

Ransom, J.I., Kaczensky, P., Lubow, B.C., Ganbaatar, O., Altansukh, N., 2012. A collaborative approach for estimating terrestrial wildlife abundance. Biol Conserv 153, 219-226.

Sokolow, W.E., Amarsanaa, G., Paklina, N.W., Posdnjakowa, M.K., Ratschkowskaja, E.I., Chotoluu, N., 1990. Das letzte Przewalskipferdeareal und seine geobotanische Charakteristik. 5. Internationales Symposium zur Erhaltung des Przewalskipferdes, Zoologischer Garten Leibzig, 214-218.

**Supplementary Information S3:** Key climate variables at sample locations (for museum samples as stated on the labels) and mean annual temperature and mean annual precipitation over the entire Dzungarian Gobi. Data based on a global climate model at 3 x 5 km resolution from a 50 year time series (1950-2000; Hijmans et al. 2005). Figure generated in ArcGIS 10.1 (ESRI, Redland, CA, USA, <http://www.esri.com/>).


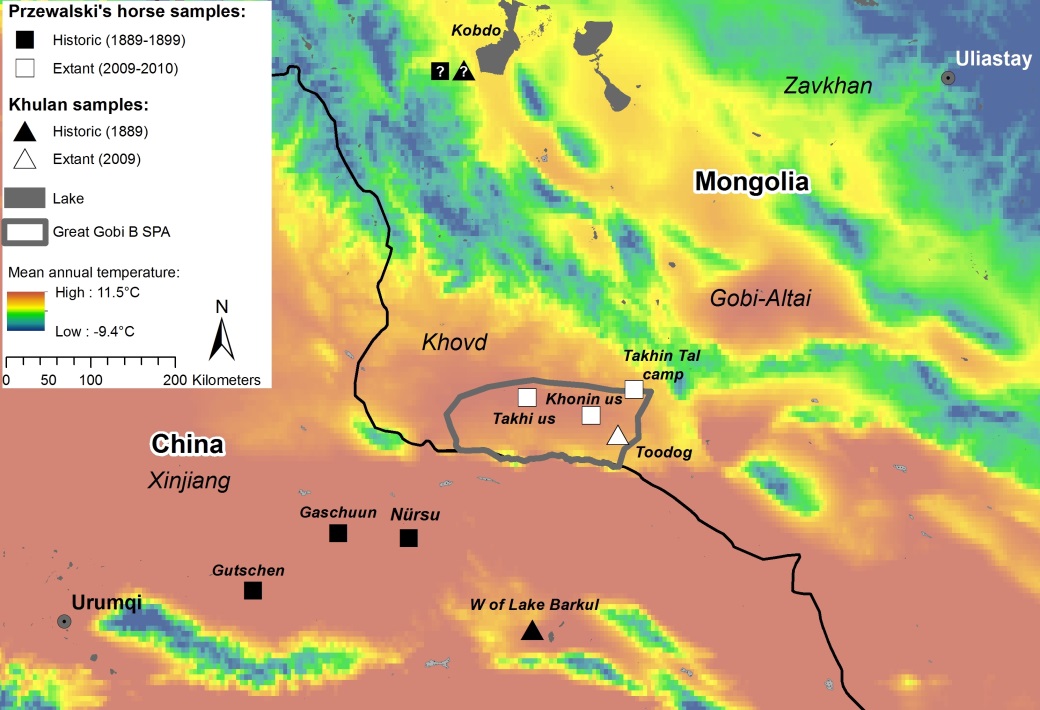

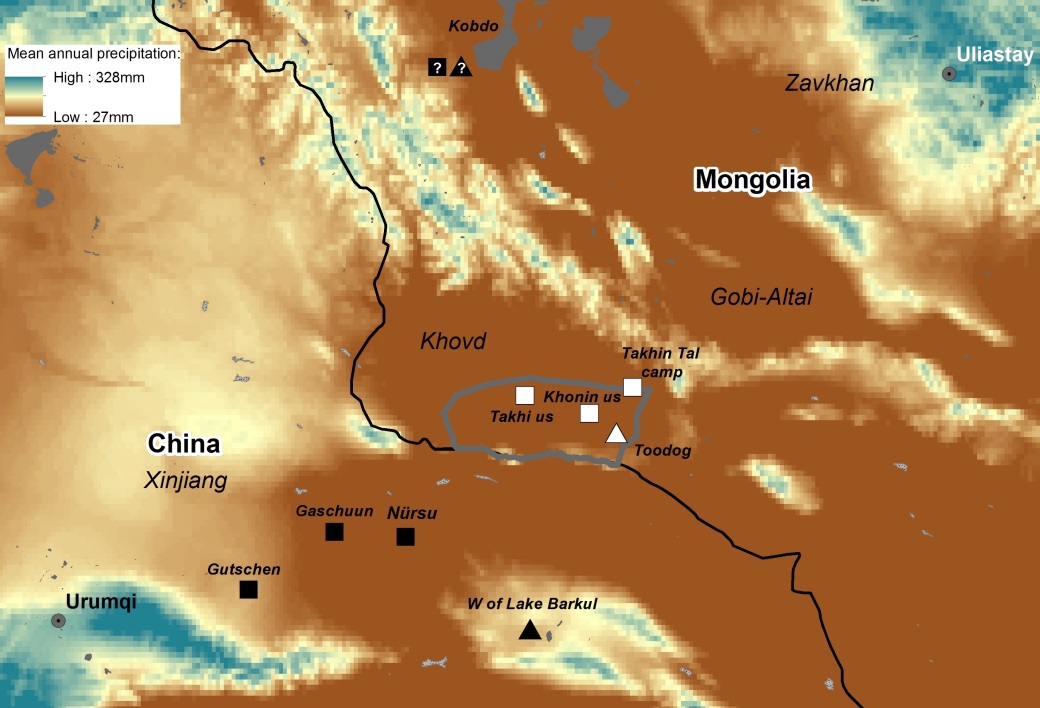


**Reference:**

Hijmans, R.J., Cameron, S.E., Parra, J.L., Jones, P.G., Jarvis, A., 2005. Very high resolution interpolated climate surfaces for global land areas. International Journal of Climatology 25, 1965-1978

**TemperaturEe**

**Precipitation**

**Supplementary Table S1:** Museum samples of historic Przewalski’s horses and khulan collected in the Dzungarian Gobi 1889-1898 and used for stable isotope analysis. RAS = Russian Academy of Sciences, MSU = Moscow State University.

| **ZIWA number scull / skin** | **Sex** | **Age estimate (years)** | **Area** | **Area represents place where animals was** | **Arrival in collection** | **Obtained in the field**** | **Fur type** | **Collector** | **Sample location** |
| --- | --- | --- | --- | --- | --- | --- | --- | --- | --- |
| ***Przewalski's horses*** | |  |  |  |  |  |  |  |  |
| 5216 / **3092** | male | 13-14  *(18)** | Gashuun | shot | 1889 | 04.09.1889 | summer | G.E. Grum-Grzhimailo & M.E. Grzhimailo | Zoological Institute, RAS, St. Petersburg |
| 5214 / **3074** | male | 9 | Gashuun | shot | 1889 | 01.09.1889 | summer | G.E. Grum-Grzhimailo & M.E. Grzhimailo | Zoological Institute, RAS, St. Petersburg |
| **1772** | female | 6 | Guchen (Qitai) | purchased | 1895 | 1895 | summer | V.I. Roborovski, P. Kozlov | Zoological Museum, MSU, Moscow |
| 5212 / **3088** | female | 10 | Nürsu | shot | 1899 | 1898 | summer | D.A. Klemenc | Zoological Institute, RAS, St. Petersburg |
| 5215 / **3087** | male | 1.5-2 *(1)** | Nürsu | shot | 1899 | 1898 | summer | D.A. Klemenc | Zoological Institute, RAS, St. Petersburg |
| 5213 / **3090***** | female | 11-12 | Kobdo district (Khovd) | purchased | 1897 | 1896 | winter | J.P. Shishmarev | Zoological Institute, RAS, St. Petersburg |
|  |  |  |  |  |  |  |  |  |  |
| ***Khulan*** |  |  |  |  |  |  |  |  |  |
| 7192 / **3062** | female | adult | W from Lake Barkul | shot | 1889 | 20.09.1889 | transition summer / winter | G.E. Grum-Grzhimailo & M.E. Grzhimailo | Zoological Institute, RAS, St. Petersburg |
| 7197 / **3066** | female | adult | W from Lake Barkul | shot | 1889 | 20.09.1889 | transition summer / winter | G.E. Grum-Grzhimailo & M.E. Grzhimailo | Zoological Institute, RAS, St. Petersburg |
| 5221 / **3079** | female | adult | Kobdo district (Khovd) | given as a present | ? | 1893-1895 | transition summer / winter | J.P. Shishmarev | Zoological Institute, RAS, St. Petersburg |

*Older estimates given in brackets, also see Grum-Grzhimailo & Grzhimailo 1896 and Garutt et al. 1966,**Based on Grum-Grzhimailo & Grzhimailo 1896 and Garutt et al. 1966, ***Originally labelled "onager" according to Salensky 1907

**References**

Garutt, E.W., Sokolow, I.I., Salesskaja, T.N., 1966. Erforschung und Zucht des Przewalski-Pferdes (*Equus przewalskii* Poljakoff) in der Sowietunion. Sonderdruck aus der "Zeitschrift für Tierzüchtung und Züchtungsbiologie" 82, 377-426.

Grum-Grzhimailo, G.E., Grzhimailo, M.E., 1896. Pages 189-211 in: Account of a journey to western China. Volume 1: Along the eastern Tian-Shan. Journal of the Russian Imperial Geographical Society, St. Petersburg, Russia. [Library of the Zoological Museum in Moscow, Russia]

Salensky, W., 1907. Prjevalsky's horse (*Equus Prjewalskii* Pol.). Hurst and Blackett Limited. <https://ia801408.us.archive.org/13/items/prjevalskyshorse00zale/prjevalskyshorse00zale.pdf>

**Supplementary Information S4:** Genetic analysis of historic Przewalski’s horse samples.

**DNA extraction and amplification**

Total genomic DNA was extracted from the tail hair portions of six historic Przewalski’s horses (*Equus (ferus) przewalskii*) following the protocol of De Volo *et al*. (2008) with slight modifications specific for dealing with hair material. After an overnight incubation at 56°C in proteinase K extraction buffer, 233 μl of 7.5 M ammonium acetate was added to each extract, vortexed for 20 sec, placed in an ice bath for 5 min and then centrifuged at 16 000 rpm for 10 min. The resulting supernatant was transferred to new tubes containing 600 μl 100% isopropanol and then 1 μl glycogen was added to each extract. Extracts were mixed by inverting tubes 50 times and were then placed in a freezer (–20°C) overnight. DNA was pelleted by centrifugation at 16 000 rpm for 10 minutes and then the isopropanol was removed before rinsing with 70% EtOH and drying by incubation at 37°C with the tubes lids open. DNA was resuspended in 20 μl AE buffer. Extracts were stored at -20°C until required for downstream analyses.

To determine the species origin of the hair samples, a 486 basepair section of mtDNA control region DNA was amplified using primers designed from published sequences. Due to the degraded nature of the extracted DNA, three separate primer pairs were designed to amplify overlapping sub-fragments of the entire 486 bp segment. The primer pairs and fragment lengths are given in Table 1. The combined fragment contains multiple polymorphic sites that differentiate domestic horse breeds (*Equus caballus*; including Mongolian domestic horses) from Przewalski’s horses as well as a 28 bp indel that is diagnostic for khulan (*Equus hemionus*). Primers were designed by eye and checked for annealing temperature suitability and potential for dimerization using the software Netprimer (Premier Biosoft). Amplification reactions were carried out in 25 μl volumes and contained 1x PCR buffer (Solis BioDyne, Estonia), 200mM each dNTP, 0.2 μM of each primer and 2 units of FIREPol™ *Taq* polymerase (Solis BioDyne, Estonia). Samples were first denatured for five minutes at 94°C followed by 35 cycles of 95°C for 30 sec, 60°C for 45 sec and 72°C for 45 sec and a final extension step of 72°C for 10 mins.

**Sequencing and data analysis**

Sequencing was performed using the Big Dye sequencing kit 3.1 (Applied Biosystems) on an ABI 3130xl. The resulting mtDNA sequences were then aligned against published sequences for Przewalski’s horses (Ishida et al. 1995, Jansen et al. 2002) and sequences retrieved from Genbank for all horse breeds including Mongolian domestic stock as well as khulan. Evolutionary relationships were reconstructed using the maximum likelihood method based on the Tamura-Nei model of nucleotide substitution (Tamura et al. 2011) and implemented in the program MEGA. The TN93 model was selected as best fitting the data based on the AIC implemented via the program jModeltest (Posada 2008).

*Supplementary Information S4 - Table 1: Primer sequences used to amplify a 486 basepair portion of the mitochondrial control region in DNA samples extracted from Equus przewalskii* hair. Three overlapping fragments were designed to amplify the entire target region. Primer binding positions are given relative to their alignment with the reference “Bonnette” haplotype (acc. # KT368749).

| **Primer Name** | **Sequence** | **Position** | **Fragment Length** |
| --- | --- | --- | --- |
| **Eprz_Dloop_1_F** | AACGTTTCCTCCCAAGGA | 15380 - 15396 |  |
| **Eprz_Dloop_1_R** | GATATTGCATGTCAGGTGGGT | 15562 - 15542 | 174bp |
| **Eprz_Dloop_2_F** | GTCAGTATCAGATTATACCCCC | 15508 - 15529 |  |
| **Eprz_Dloop_2_R** | TGGAACATGGGTTGTGATATG | 15728 - 15708 | 221bp |
| **Eprz_Dloop_3_F** | TCCAAGTCAAATCATTTCCA | 15679 - 15698 |  |
| **Eprz_Dloop_3_R** | AATGGCCCTGAAGAAAGAAC | 15865 - 15846 | 187bp |

**Results**

Our historical samples 3088, 3087, and one extant Przewalski’s horse control sample (13-057) were identical with the Przewalski’s horse haplotype “Bijsk” (accession number KT368753, E value = 0.0) and samples 3090, 3074, and 3092 were identical with the Przewalski’s horse haplotype “Bonnette” (accession number KT368749, E value = 0.0). Historic sample 1772 matched a domestic horse sequence (accession number LN398425, E value 0.0).

A phylogram representing the relationships between different horse mtDNA control region sequences is shown below (Supplementary Information S4 - Figure 1). The published Przewalski’s sequences are placed in separate sections of the tree representing the mixed founding stock of Przewalski’s horses.

**Summary and conclusion**

The mtDNA analyses conducted leave no doubt that all of the sequences represent DNA extracted from horses and not khulan. Five sequences further confirmed Przewalski’s horse stock, though the sequence of sample 1772 likely comes from a domestic horse mother, implying a certain degree of genetic introgression.

Introgression of domestic genes into the wild Przewalski’s horse genepool prior to extinction in the wild has been previously demonstrated and as it seems to have been primarily female mediated mtDNA analysis *per se* is not diagnostic for species assignment (Ishida et al. 1995; Goto et al. 2011).

Sample 1772 originates from a mounted specimen at the Zoological Museum in Moscow which shows the phenotypical characteristics of a Przewalski’s horse (Salensky 1907; Supplementary Information S4 - Figure 2), making it unlikely to be a first-generation hybrid, but rather suggests subsequent back-crossing and life among Przewalski’s horses. Consequently, there is little doubt about the assignment of all six historic samples as originating from free-ranging, wild Przewalski’s horses.

Museum samples used for isotope analysis

Extant Przewalski’s horse positive control

Published Przewalski’s horse sequences PH1-PH7

Domestic horse sequences of Mongolian origin

Domestic horse sequences of European origin

Known domestic / Przewalski’s horse hybrid sequences

Asiatic wild ass samples

**DH1-DH20:**

**DH21-DH26:**

**Phyb1-Phyb5:**

**KH1-KH4:**

*Supplementary Information S4 - Figure 1: Maximum Likelihood analysis of project samples in relation to published Equid (Equus sp) sequences.*

All corresponding accession numbers are given below in Supplementary Information S4 - Table 2 for published sequences and in Supplementary Table S2 for our museum sequences. The evolutionary history was inferred using the Maximum Likelihood method based on the Kimura 2-parameter model (Kimura 1980). The tree with the highest log likelihood (-957.8889) is shown. The percentage of trees in which the associated taxa clustered together is shown next to the branches (only values over 50% shown). Initial tree(s) for the heuristic search were obtained automatically by applying Neighbor-Join and BioNJ algorithms to a matrix of pairwise distances estimated using the Maximum Composite Likelihood (MCL) approach, and then selecting the topology with superior log likelihood value. A discrete Gamma distribution was used to model evolutionary rate differences among sites (5 categories (+*G*, parameter = 0.2161)). The tree is drawn to scale, with branch lengths measured in the number of substitutions per site. The analysis involved 50 nucleotide sequences. All positions containing gaps and missing data were eliminated. There were a total of 293 positions in the final dataset. Evolutionary analyses were conducted in MEGA7 (Kumar et al. 2016).

*Supplementary Information S4 -* Table 2: Equus sequences used in this study for phylogeny construction and determination of sample origin.

| **Code** | **Source** | **Accession #** |
| --- | --- | --- |
| DH1 | Mongolian Domestic | DQ297632 |
| DH2 | Mongolian Domestic | AF014415 |
| DH3 | Mongolian Domestic | DQ297623 |
| DH4 | Mongolian Domestic | AF056071 |
| DH5 | Mongolian Domestic | DQ297633 |
| DH6 | Mongolian Domestic | DQ297629 |
| DH7 | Mongolian Domestic | AF014414 |
| DH8 | Mongolian Domestic | DQ297630 |
| DH9 | Mongolian Domestic | AF014413 |
| DH10 | Mongolian Domestic | DQ297625 |
| DH11 | Mongolian Domestic | DQ297635 |
| DH12 | Mongolian Domestic | AF014405 |
| DH13 | Mongolian Domestic | DQ297624 |
| DH14 | Mongolian Domestic | DQ297626 |
| DH15 | Mongolian Domestic | DQ297638 |
| DH16 | Mongolian Domestic | DQ297637 |
| DH17 | Mongolian Domestic | DQ297631 |
| DH18 | Mongolian Domestic | DQ297627 |
| DH19 | Mongolian Domestic | DQ297628 |
| DH20 | Mongolian Domestic | DQ297622 |
| DH21 | Iranian Domestic | JN398423 |
| DH22 | German Domestic | JN398429 |
| DH23 | Mongolian Domestic | KT211156 |
| DH24 | Mongolian Domestic | GU561997 |
| DH25 | Asian Domestic | JN398393 |
| DH26 | American Domestic | JN398377 |
| KH1 | Khulan1 | KP825312 |
| KH2 | Khulan2 | AF220936 |
| KH3 | Khulan3 | AF220935 |
| KH4 | Khulan4 | KP825311 |
| PH1 | Przewalski᾽s horse isolate *Kolette* | KT368752 |
| PH2 | Przewalski᾽s horse isolate *Bogatka* | AF055878 |
| PH3 | Przewalski᾽s horse Holotype | KT368755 |
| PH4 | Przewalski᾽s horse isolate *Bonnette* | KT368749 |
| PH5 | Przewalski᾽s horse isolate *Bellina* | AP012267 |
| PH6 | Przewalski᾽s horse isolate *Bijsk* | KT368753 |
| PH7 | Przewalski᾽s horse isolate *Bars* | AP012270 |
| Phyb1 | Przewalski᾽s horse hybrid *Viola* | KT368742 |
| Phyb2 | Przewalski᾽s horse hybrid *Vjuga* | KT368743 |
| Phyb3 | Przewalski᾽s /Mongolian hybrid | unpublished sequence |
| Phyb4 | Przewalski᾽s horse hybrid *Theodor* | KT368758 |
| Phyb5 | Przewalski᾽s horse hybrid KB7903 | KT368757 |
| 1772 | Hair sample - Zoological Museum, MSU, Moscow | This study |
| 3074 | Hair sample - Zoological Institute, RAS, St. Petersburg | This study |
| 3087 | Hair sample - Zoological Institute, RAS, St. Petersburg | This study |
| 3088 | Hair sample - Zoological Institute, RAS, St. Petersburg | This study |
| 3090 | Hair sample - Zoological Institute, RAS, St. Petersburg | This study |
| 3092 | Hair sample - Zoological Institute, RAS, St. Petersburg | This study |
| 13_057 | Hair sample from Takhin Tal, Mongolia (EEP number 5341; mare *Tsaganaa*) | This study |
| Donkey | Outgroup | X97337 |

RAS = Russian Academy of Sciences, MSU = Moscow State University


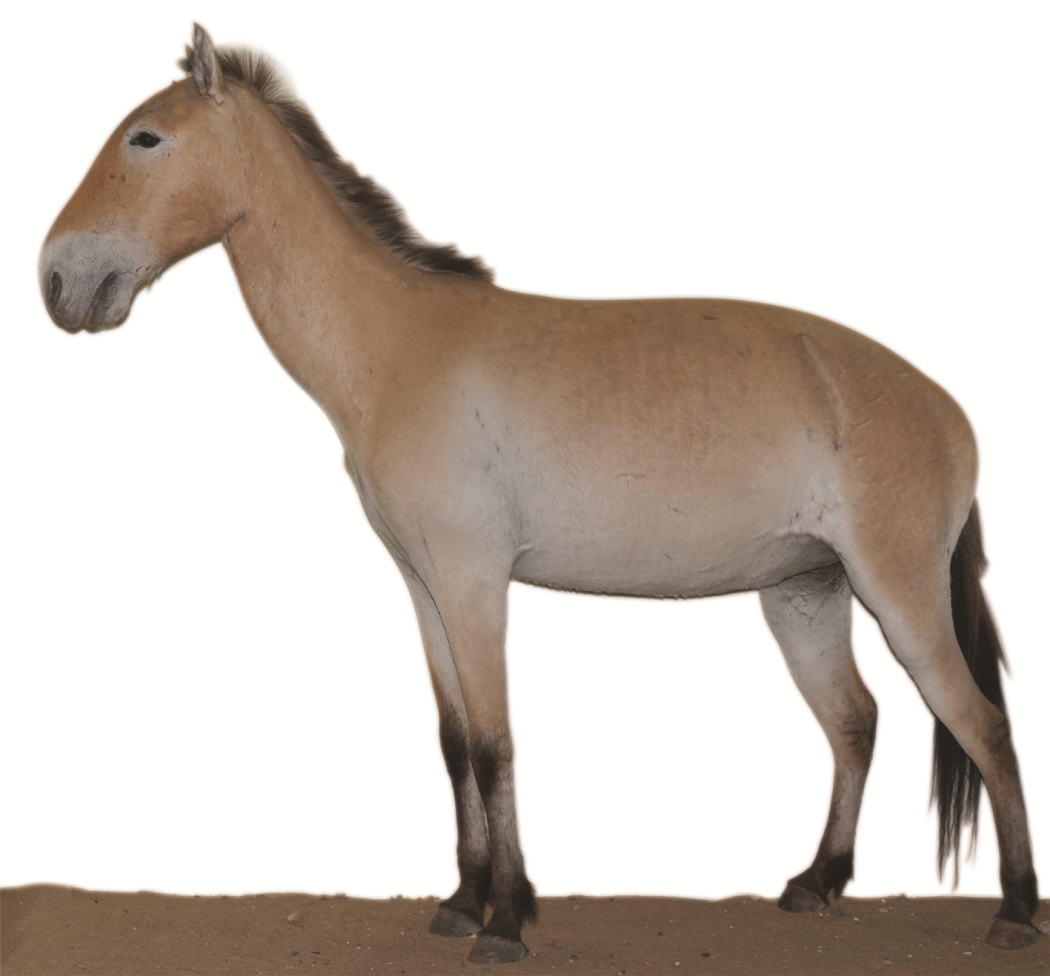
*Supplementary Information S4 - Figure 2: Sample 1772 on display at the Zoological Museum in Moscow, Russia. Photo: N.N. Spasskaya*

**References**

De Volo, S.B., Reynolds, R.T., Douglas, M.R., Antolin, M.F., 2008. An improved extraction method to increase DNA yield from molted feathers. The Condor 110, 762-766.

Posada, D., 2008. jModelTest: Phylogenetic model averaging. Mol Biol Evol 25, 1253-1256.

Ishida, N., Oyunsuren, T., Mashima, S., Mukoyama, H., Saitou, N., 1995. Mitochondrial DNA sequences of various species of the genus *Equus*; with special reference to the phylogenetic relationship between Przewalskii's wild horse and domestic horse. Journal of Molecular Evolution 41, 180-188.

Jansen, T., Forster, P., Levine, M.A., Oelke, H., Hurles, M., Renfrew, C., Weber, J.r., Olek, K., 2002. Mitochondrial DNA and the origins of the domestic horse. Proc Natl Acad Sci U S A 99, 10905-10910.

Tamura, K., Peterson, D., Peterson, N., Stecher, G., Nei, M., Kumar, S., 2011. MEGA5: Molecular evolutionary genetics analysis using maximum likelihood, evolutionary distance, and maximum parsimony methods. Mol Biol Evol 28, 2731-2739.

Goto, H., Ryder, O.A., Fisher, A.R., Schultz, B., Kosakovsky Pond, S.L., Nekrutenko, A., Makova, K.D., 2011. A massively parallel sequencing approach uncovers ancient origins and high genetic variability of endangered Przewalski's horses. Genome Biol Evol 3, 1096-1106.

Salensky, W., 1907. Prjevalsky's horse (*Equus Prjewalskii* Pol.). Hurst and Blackett Limited, London, UK. <https://ia801408.us.archive.org/13/items/prjevalskyshorse00zale/prjevalskyshorse00zale.pdf>

Kimura M., 1980. A simple method for estimating evolutionary rates of base substitutions through comparative studies of nucleotide sequences. Journal of Molecular Evolution 16, 111-120.

Kumar S., Stecher G., Tamura K., 2016. MEGA7: Molecular Evolutionary Genetics Analysis version 7.0 for bigger datasets. Molecular Biology and Evolution, msw054.

**Supplementary Table S2:** Mean stable isotope ratios of tail hair samples of historic and extant Przewalski’s horses and khulan collected in the Dzungarian Gobi from 1889-1898 (historic, individual values) and 2009-2010 (extant, pooled values only), respectively.

| **ZIWA number skull / skin** | **Sex** | **Age (years)** | ***δ*^13^C_hair_ (‰)** | | | |  |  | ***δ*^15^N_hair_ (‰)** | | | |  |  | ***δ*^2^H_hair_ (‰)** | | | |  |
| --- | --- | --- | --- | --- | --- | --- | --- | --- | --- | --- | --- | --- | --- | --- | --- | --- | --- | --- | --- |
|  |  |  | **Mean** | **SD** | **Min** | **Max** | **N** |  | **Mean** | **SD** | **Min** | **Max** | **N** |  | **Mean** | **SD** | **Min** | **Max** | **N** |
| ***Przewalski᾽s horse*** |  |  |  |  |  |  |  |  |  |  |  |  |  |  |  |  |  |  |  |
| 5216 / **3092** | male | 13-14 | -20.7 | 1.3 | -22.9 | -18.8 | 33 |  | 11.9 | 1.6 | 8.2 | 13.9 | 33 |  | -143 | 10 | -172 | -122 | 31 |
| 5214 / **3074** | male | 9 | -20.9 | 1.3 | -23.0 | -18.9 | 20 |  | 11.6 | 1.9 | 8.9 | 13.8 | 20 |  | -139 | 13 | -166 | -118 | 16 |
| **1772** | female | 6 | -20.6 | 2.9 | -24.4 | -14.8 | 49 |  | 10.5 | 0.6 | 9.2 | 12.2 | 49 |  | -140 | 9 | -152 | -113 | 34 |
| 5212 / **3088** | female | 10 | -20.1 | 2.1 | -23.1 | -14.8 | 31 |  | 10.9 | 1.5 | 8.5 | 13.8 | 31 |  | -151 | 16 | -188 | -123 | 23 |
| 5215 / **3087** | male | 1.5-2 | -20.0 | 2.0 | -22.7 | -15.0 | 35 |  | 12.2 | 1.7 | 9.8 | 14.8 | 35 |  | n.a. | n.a. | n.a. | n.a. | 0 |
| 5213 / **3090** | female | 11-12 | -23.1 | 0.5 | -24.0 | -22.4 | 22 |  | 10.9 | 1.5 | 9.1 | 13.4 | 22 |  | -155 | 9 | -170 | -135 | 16 |
| ****mean [sum] historic*** | |  | ***-20.7*** | ***2.2*** | ***-24.4*** | ***-14.8*** | ***[190]*** |  | ***11.3*** | ***1.6*** | ***8.2*** | ***14.8*** | ***[190]*** |  | ***-145*** | ***13*** | ***-188*** | ***-113*** | ***[120]*** |
| ****mean [sum] extant (N=6)*** | | | ***-23.4*** | ***0.9*** | ***-25.2*** | ***-18.9*** | ***[308]*** |  | ***8.2*** | ***1.8*** | ***3.8*** | ***12.5*** | ***[308]*** |  | ***-151*** | ***20*** | ***-197*** | ***-111*** | ***[291]*** |
|  |  |  |  |  |  |  |  |  |  |  |  |  |  |  |  |  |  |  |  |
| ***Khulan*** |  |  |  |  |  |  |  |  |  |  |  |  |  |  |  |  |  |  |  |
| 7192 / **3062** | female | adult | -21.8 | 1.8 | -24.0 | -17.4 | 46 |  | 10.0 | 0.6 | 8.7 | 11.1 | 46 |  | -139 | 14 | -170 | -114 | 42 |
| 7197 / **3066** | female | adult | -21.5 | 1.6 | -23.6 | -17.6 | 63 |  | 9.2 | 1.0 | 7.1 | 11.6 | 63 |  | -134 | 13 | -161 | -111 | 57 |
| 5221 / **3079** | female | adult | -21.8 | 1.7 | -23.5 | -17.0 | 32 |  | 11.1 | 0.8 | 9.8 | 12.5 | 32 |  | -134 | 17 | -163 | -112 | 27 |
| ****mean [sum] historic*** | |  | ***-21.7*** | ***1.7*** | ***-24.0*** | ***-17.0*** | ***[141]*** |  | ***9.9*** | ***1.1*** | ***7.1*** | ***12.5*** | ***[141]*** |  | ***-136*** | ***15*** | ***-170*** | ***-111*** | ***[126]*** |
| ****mean [sum] extant (N=6)*** | | | ***-21.2*** | ***1.7*** | ***-24.2*** | ***-17.4*** | ***[275]*** |  | ***8.7*** | ***1.0*** | ***6.9*** | ***12.9*** | ***[275]*** |  | ***-130*** | ***16*** | ***-178*** | ***-94*** | ***[271]*** |
| *mean and sum of all increments | | |  |  |  |  |  |  |  |  |  |  |  |  |  |  |  |  |  |

***Supplementary Fig. S1:*** *Carbon isotope (δ^13^C_diet_ ) profiles of extant and historic khulan in the Dzungarian Gobi. Photo: P. Kaczensky*


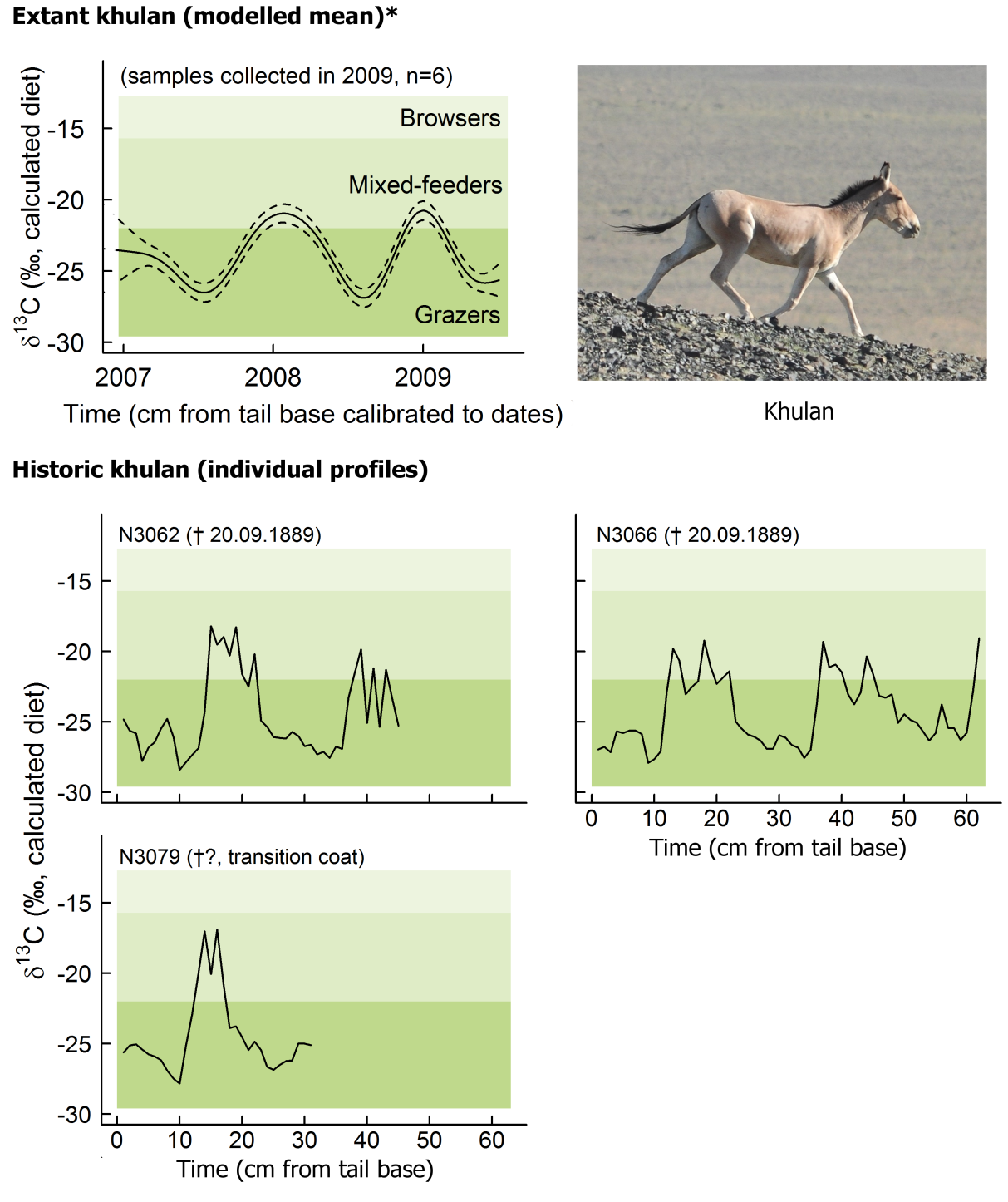


**Supplementary Fig. S2:** Size of the core isotopic dietary niches expressed as the area of ellipses (‰^2^) in the two-dimensional isotopic space of reintroduced, extant and historic Przewalski’s horses and sympatric historic and extant khulan in the Dzungarian Gobi. Black dots represent the mode, gray dots are true population values and the shaded boxes represent the 50%, 75% and 95% credible intervals from dark to light grey. Artwork: M. van Dalum.


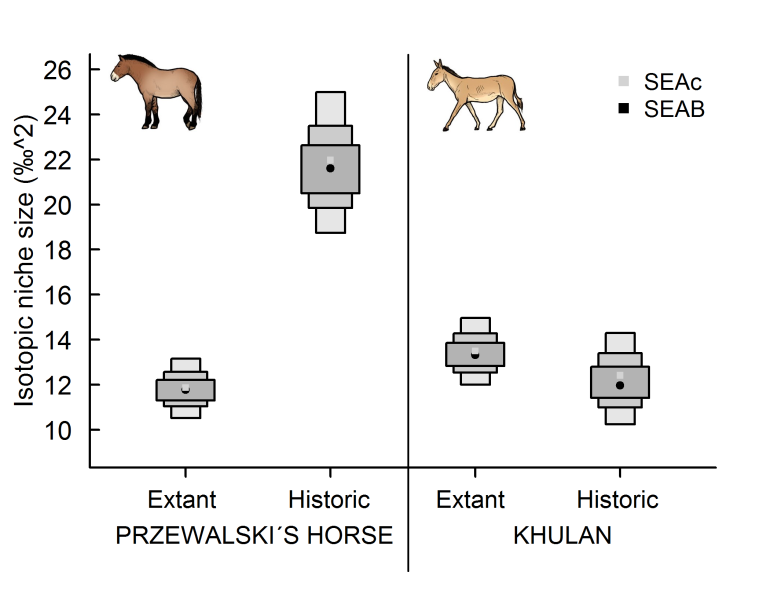


***Supplementary Fig. S3:*** *Individual isotopic dietary niches of six historic and six extant Przewalski’s horses in the Dzungarian Gobi. a) Shape, and b) Size, expressed as the area of ellipses (‰^2^) in the two-dimensional isotopic space. Black dots represent the mode, gray dots are true population values and the shaded boxes represent the 50%, 75% and 95% credible intervals from dark to light grey. Artwork: M. van Dalum.*


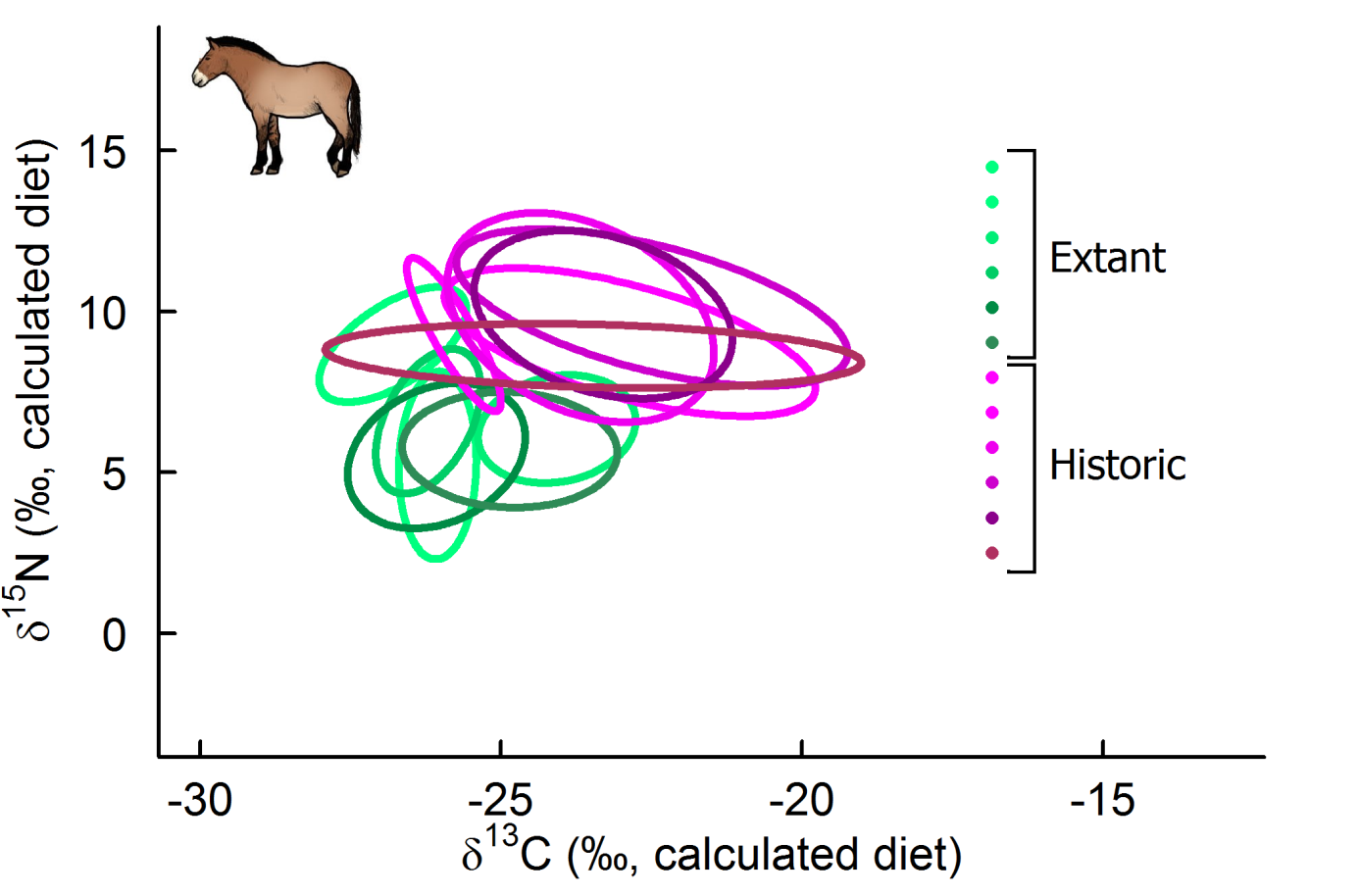
**a)**

**b)**


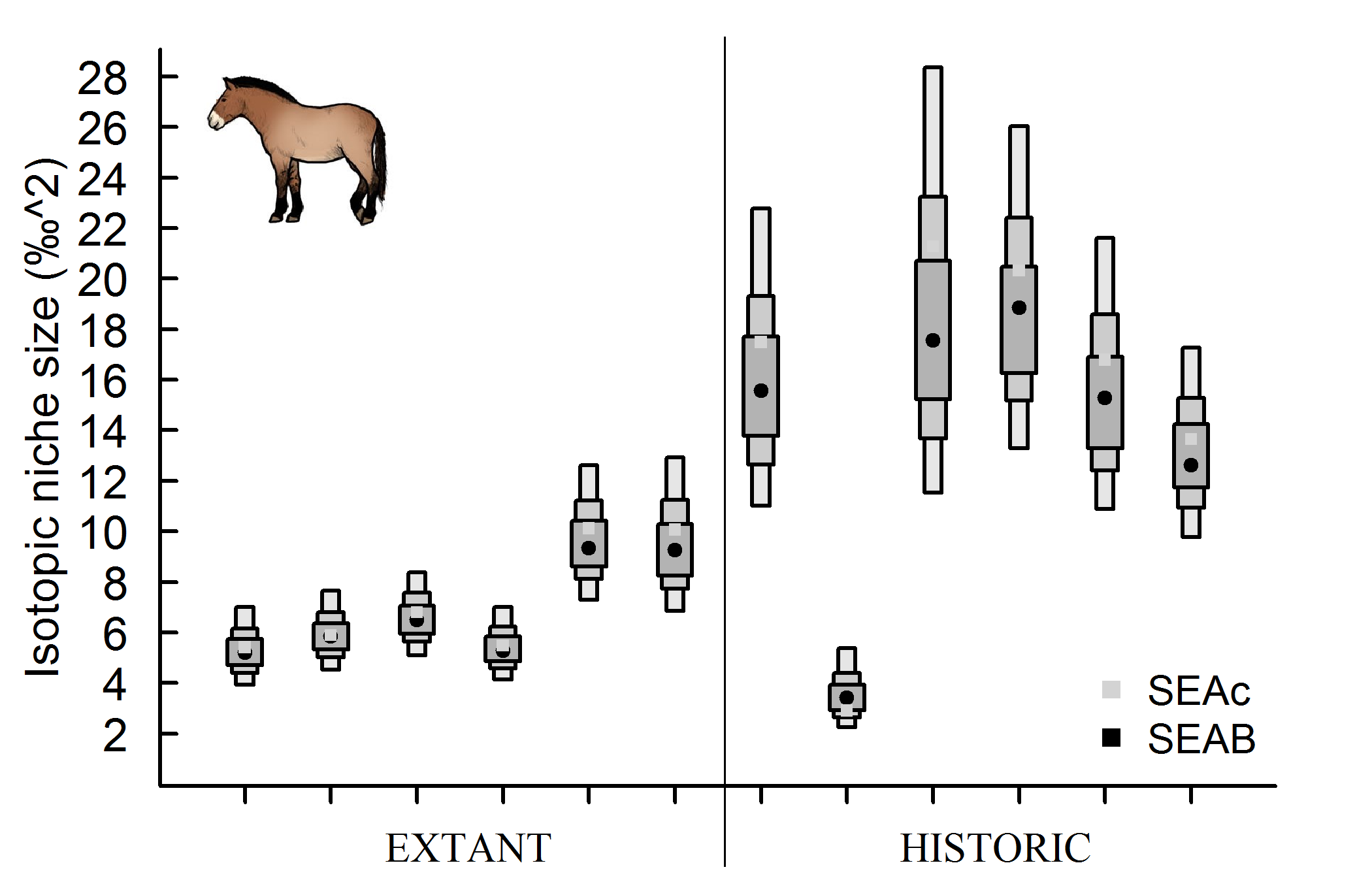


**Supplementary Fig. S4:** Individual core isotopic dietary niches of three historic and six extant khulan in the Dzungarian Gobi. a) Shape, and b) Size, expressed as the area of ellipses (‰^2^) in the two-dimensional isotopic space. Black dots represent the mode, gray dots are true population values and the shaded boxes represent the 50%, 75% and 95% credible intervals from dark to light grey. Artwork: M. van Dalum.


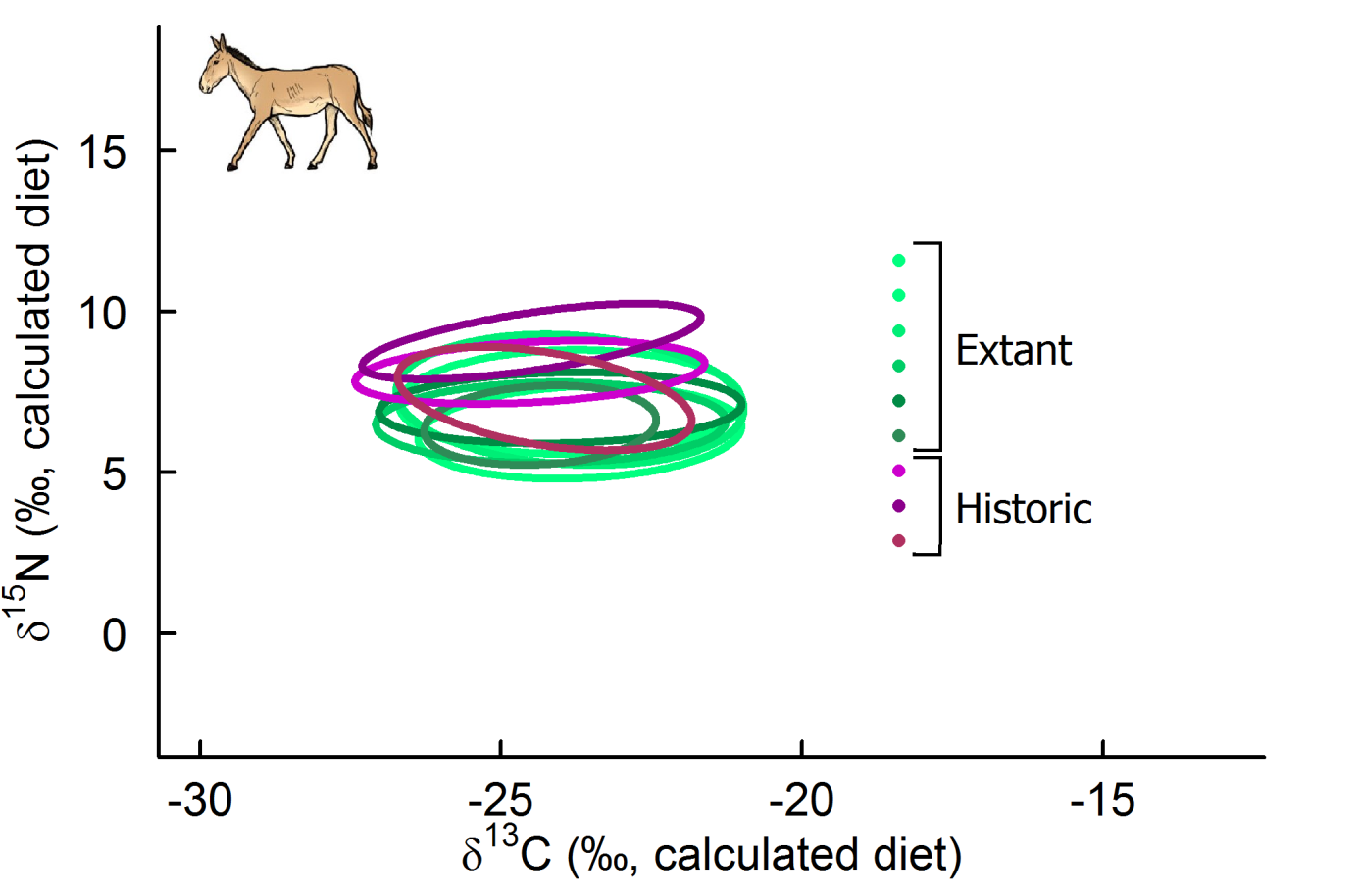
**a)**


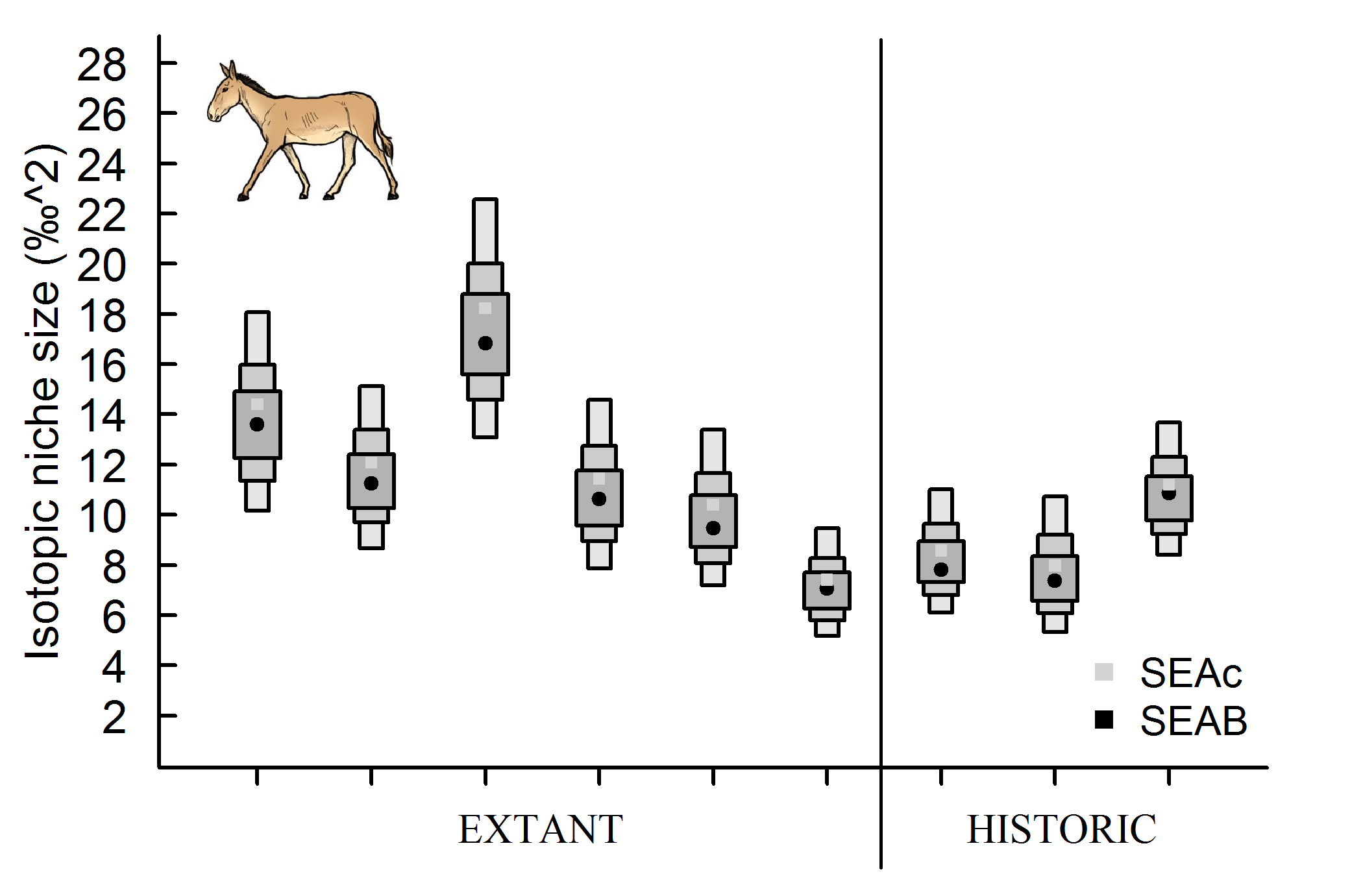


**b)**

***Supplementary Information S5:*** *References for figure 5: Living conditions for historic and extant Przewalski’s horses and khulan.*

***Przewalski’s horse*** [*magnitude of population size in 1918 inferred from range, number of animals observed, and number of foals captured for transports to Europe*]:

Bouman, I., Bouman, J., 1994. The history of Przewalski’s Horse. Pages 5-38 in L.Boyd and D.A. Houpt (Eds). Przewalski’s horse -The History and Biology of an Endangered Species. State University of New York Press, Albany.

Grum-Grzhimailo, G.E., Grzhimailo, M.E., 1896. Pages 189-211 in: Account of a journey to western China. Volume 1: Along the eastern Tian-Shan. Journal of the Russian Imperial Geographical Society, St. Petersburg, Russia. [Library of the Zoological Museum in Moscow, Russia]

Kaczensky, P., Hrabar, H., Lukarevski, V., Zimmermann, W., Usukhjargal, D., Ganbaatar, O., Bouskila, A., 2016. Reintroduction of Wild Equids. Pages 196-214 in: Ransom, J.I., and Kaczensky, P. (Eds). Wild Equids - Ecology, Management, and Conservation. Johns Hopkins University Press, Baltimore, USA.

King, S.R.B., Boyd, L., Zimmermann, W., Kendall, B.E., 2015. *Equus ferus*. The IUCN Red List of Threatened Species 2015: e.T41763A45172856.

Volf, J., 1996. Das Urwildpferd. Die Neue Brehm Bücherei, Band 249, Westarp Wissenschaften, Magdeburg, Germany. [in German]

Zevegmid, D., Dawaa, N., 1973. Die seltenen Großsäuger der Mongolischen Volksrepublik und ihr Schutz. Archiv für Naturschutz und Landschaftsforschung 13, 87-106. [in German]

***Khulan*** [*magnitude of population size in 1918 inferred from range reduction and current population densities in Mongolia*]:

Bannikov, A.G., 1981. The Asian Wild Ass. Lesnaya Promyshlennost, Moscow, Russia. [original in Russian, English translation by M. Proutkina, Zoological Society of San Diego].

Buuveibaatar, B., Strindberg, S., Kaczensky, P., Payne, J., Chimeddorj, B., Naranbaatar, G., Amarsaikhan, S., Dashnyam, B., Munkhzul, T., Purevsuren, T., Hosack, D.A., Fuller, T.K., 2016 EarlyOnline. Mongolian Gobi supports the world’s largest populations of khulan and goitered gazelles. Oryx. DOI: 10.1017/S0030605316000417

Kaczensky, P., Kuehn, R., Lhagvasuren, B., Pietsch, S., Yang, W., Walzer, C., 2011. Connectivity of the Asiatic wild ass population in the Mongolian Gobi. Biol Conserv 144, 920-929.

Kaczensky, P., Lkhagvasuren, B., Pereladova, O., Hemami, M.-R., Bouskila, A., 2015. Equus hemionus. The IUCN Red List of Threatened Species 2015: e.T7951A45171204.

Ransom, J.I., Kaczensky, P., Lubow, B.C., Ganbaatar, O., Altansukh, N., 2012. A collaborative approach for estimating terrestrial wildlife abundance. Biol Conserv 153, 219-226.

Reading, R.P., Mix, H.M., Lhagvasuren, B., Feh, C., Kane, D.P., Dulamtseren, S., Enkhbold, S., 2001. Status and distribution of khulan (*Equus hemionus*) in Mongolia. Journal of Zoology, London 254, 381-389.

**Livestock and human population:**

Mongolian Statistical Information Service - Livestock. <http://www.1212.mn/en/> [*see Number of livestock, by type, by region, soums, aimags and the capital – 2013*]

Neupert, R., Tsogtsaikhan, B., Byambaa, E., Tumur, G., Tumertolgoi, N., Chimedtseren, T., Hayes, G., 2012. Implications of demographic trends for socio-economic development and public policy in Mongolia. United Nations Population Fund, Ulaanbaatar, Mongolia.[*for human population in 1918 see Table 1.1*]; [http://unfpa.org.mn/Demographic%20study%20English.pdf](http://unfpa.org.mn/Demographic study English.pdf)

Suttie, J.M., 2005. Grazing management in Mongolia. Pages 265-304 in Suttie, J. M., Reynolds, S. G. & Batello, C. (Eds). Grassland of the world. Plant Production and Protection Series, No. 34, Food and Agricultural Organization (FAO) of the United Nations, Rome, Italy. [*for livestock numbers in 1918 see Table 7.10*]; <http://www.fao.org/docrep/008/y8344e/y8344e00.htm>
